# A nutrigenetic approach for investigating the relationship between vitamin B12 status and metabolic traits in Indonesian women

**DOI:** 10.1007/s40200-019-00424-z

**Published:** 2019-07-25

**Authors:** S. Surendran, A. S. Aji, U. Ariyasra, S. R. Sari, S. G. Malik, N. Tasrif, F. F. Yani, J. A. Lovegrove, I. R. Sudji, N. I. Lipoeto, Karani Santhanakrishnan Vimaleswaran

**Affiliations:** 1 Hugh Sinclair Unit of Human Nutrition, Department of Food and Nutritional Sciences, University of Reading, Reading, UK; 2 Department of Biomedical Science, Faculty of Medicine, Andalas University, Padang, West Sumatra Indonesia; 3 Eijkman Institute for Molecular Biology, Jakarta, Indonesia; 4 Public Health Department, Faculty of Medicine, Andalas University, Padang, Indonesia; 5 Department of Child Health, Faculty of Medicine, Andalas University, Padang, Indonesia; 6 Institute for Cardiovascular and Metabolic Research (ICMR), University of Reading, Reading, UK; 7 Biomedical Laboratory, Faculty of Medicine, Andalas University, Padang, Indonesia; 8 Department of Nutrition, Faculty of Medicine, Andalas University, Padang, Indonesia

**Keywords:** Glycated haemoglobin, Waist circumference, Metabolic traits, Vitamin B12 pathway, Indonesian, Nutrigenetics

## Abstract

**Purpose:**

Adverse effects of maternal vitamin B12 deficiency have been linked to major clinical outcomes, including increased body mass index and gestational diabetes, however, less is known about vitamin B12 nutrition in non-pregnant women. Hence, the aim of the present study was to explore the relationships between metabolic traits and vitamin B12 status in a cohort of healthy Indonesian women and to investigate whether these relationships were modified by dietary intake using a genetic approach.

**Methods:**

A total of 117 Minangkabau women (aged 25–60 years), from the city of Padang, West Sumatra underwent anthropometric, biochemical, dietary intake analysis and genetic tests. Genetic risk scores (GRS) based on nine vitamin B12 associated single nucleotide polymorphisms (SNPs) (B12-GRS) and nine metabolic SNPs (metabolic-GRS) were constructed.

**Results:**

The B12-GRS and metabolic-GRS had no effect on vitamin B12 (*P* > 0.160) and metabolic traits (*P* > 0.085). However, an interaction was observed between the B12-GRS and dietary fibre intake (g) on glycated haemoglobin (HbA1C) levels (P _interaction_ = 0.042), where among those who consumed a low fibre diet (4.90 ± 1.00 g/day), individuals carrying ≥9 risk alleles for vitamin B12 deficiency had significantly higher HbA1C levels (*P* = 0.025) compared to those carrying ≤8 risk alleles.

**Conclusion:**

Our study showed a significant impact of the B12-GRS on HbA1C concentrations through the influence of a dietary factor, however, our study failed to provide evidence for an impact of metabolic-GRS on lowering B12 concentrations. Further replication studies utilizing larger sample sizes are needed to confirm our findings.

**Electronic supplementary material:**

The online version of this article (10.1007/s40200-019-00424-z) contains supplementary material, which is available to authorized users.

## Introduction

Vitamin B12 adequacy plays a critical role in a multitude of physiological processes, including DNA synthesis, haematological development and neurological function [1, 2]. Moreover, vitamin B12 is now known to play a much more profound and wide-ranging role in maternal health as well as foetal development [3, 4]. Low maternal plasma concentrations of vitamin B12 have shown negative correlations with body mass index (BMI) levels in healthy women [5] and have been associated with pregnancy complications such as gestational diabetes mellitus [3], recurrent pregnancy loss [6], higher BMI [7] and neural tube defects [8]. Notably, the harmful effects of maternal malnutrition are not just confined to pregnancy complications and birth defects. Findings from the Pune Maternal Nutrition Study (PMNS) in India have shown that low maternal vitamin B12 increases the risk of insulin resistance and relative adiposity in 6- to 7-y-old children, with the highest levels of insulin resistance occurring when mothers had a combination of a high folate and low vitamin B12 status [7].

Suboptimal vitamin B12 status has been shown to be prevalent in many countries [9]. Published data on vitamin B12 status of any life-stage group in Indonesian women is lacking with the exception of an earlier report in 2017 showing that the prevalence of a vitamin B12 deficient diet was 34.5% in 606 Indonesian pregnant women (14–49 years) [10]. The Minangkabau culture in Indonesia is of particular interest, as it is the world’s largest matrilineal system of kinship, where women hold greater power in both family and society [11]. Food supply is centred around women and compelling evidence suggests that adequate nutrition protects against metabolic disorders related to obesity [12], as a result understanding the dietary patterns of women in relation to their genetic susceptibility is of great importance.

Although vitamin B12 deficiency is associated with a wide range of chronic diseases and conditions, including obesity, and with increasing severity of metabolic dysfunction, such as insulin dysregulation [13–16], the relationship between low vitamin B12 status and obesity related traits has remained inconsistent [17]. It is possible that certain genotypes might jointly contribute to obesity and vitamin B12 deficiency [17] and the implementation of a genetic approach to establish the relationship between vitamin B12 and obesity could be a more desirable option over observational studies, as results are less prone to confounding factors. While genetic studies have implicated several gene loci in the predisposition to vitamin B12 deficiency, no study has yet been carried out in the Indonesian population [18]. Hence, for the first time we used a genetic approach to explore the relationship between metabolic traits and vitamin B12 status and investigated whether these relationships were modified by lifestyle factors in a cohort of Minangkabau women in Padang. Identifying the impact of vitamin B12 status on metabolic traits will help us to reduce the burden of metabolic diseases through implementation of policies for screening of vitamin B12 deficiency.

## Methodology

**Fig. 1:**
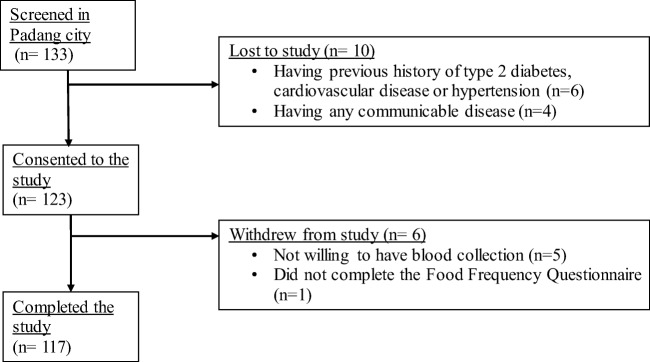
Flow chart of the subject recruitment process

The Minangkabau Indonesia Study on Nutrition and Genetics (MINANG) study is a cross-sectional pilot study that was conducted in the city of Padang, West Sumatra, Indonesia, between December 2017 to January 2018. This study was conducted as part of the ongoing **GeNuIne** (**Ge**ne-**Nu**trient **In**t**e**ractions) Collaboration, the main objective of which is to investigate the effect of gene-nutrient interactions (nutrigenetics) on metabolic disease outcomes using population based studies from various ethnic groups [19]. The study was conducted in accordance with the principles of the Declaration of Helsinki and was approved by the Ethical Review Committee of the Medical Faculty, Andalas Univesity (No.311/KEP/FK/2017). All participants provided written informed consent before participating. Participants were allowed to leave the study at will and opt out from any of the procedures. One hundred and thirty-three women were recruited from community health centers in two sub districts in Padang City to represent both urban (50% Padang Timur) and rural (50% Kuranji) areas of Padang population. Inclusion criteria were healthy women (between 25 and 60 years old) with Minangkabau ethnicity. Among the 133 eligible adults, 10 adults were excluded from the study. Exclusion criteria included the following: having a previous history of type 2 diabetes, cardiovascular disease or hypertension (*n* = 6), having a BMI of more than 40 kg/m^2^ or being classified as morbidly obese by a physician (*n* = 0), being blood related to other participants in the study (n = 0), having any communicable disease (*n* = 4), being pregnant or lactating (n = 0) and taking dietary or vitamin supplements (n = 0). Among the 123 remaining adults, 5 volunteers did not undergo blood sample collection and were excluded from the study and one participant did not undertake the validated semi-quantitative food frequency questionnaire (FFQ) [20]. The final sample consisted of 117 women who completed a FFQ and underwent blood sample collection for biochemical and genetic analysis (Fig. 1).

### Anthropometric measures

Body weight was measured to the nearest 100 g using an electronic scale (Seca 803, Seca GmbH. Co. kg, Hamburg, Germany) and height was measured to the nearest mm using a stadiometer (OneMed Medicom stature meter, YF.05.05.V.A.1022, Indonesia). The BMI was estimated as weight (kg) divided by the height (m) squared. BMI was classified according to the Asia-Pacific classification for BMI/age according to sex [21]. The waist (cm) circumference was measured in a standing position with the feet positioned close together. The waist circumference (WC) was measured using a metal tape (Medline-OneMed Medicom, Jakarta, Indonesia) midway between the lower border of the rib cage and the iliac crest, at the end of gentle expiration. Body fat percentage was measured using the Tanita MC780 multi frequency segmental body composition analyser.

### Biochemical measures

For the determination of biochemical parameters, blood samples (5 ml) were collected by a trained phlebotomist in the morning, after a 12 h fast. The blood samples were used to measure vitamin B12, glucose, insulin and glycated hemoglobin (HbA1c). All biochemical samples were assayed using the xMark Microplate Spectrophotometer (Bio-Rad Laboratories Inc., Hercules, California, USA). Serum concentrations of vitamin B12, glucose, insulin and HbA1c were assessed using enzyme-linked immunosorbent assay (ELISA) kits from Bioassay Technology Laboratory (Shanghai, China).

### Assessment of dietary intake and physical activity

Data collection was completed by a qualified nutritionist in the home or in an integrated health service post. Dietary intakes were assessed using a previously validated and published semi-quantitative food frequency questionnaire (SQ-FFQ) containing 223 food items [20]. In brief, participants were asked to estimate the usual frequency (number of times per day, week or month) and the portion sizes of various food items. Portion size photographs of all relevant foods (including some prepared dishes) were used by participants while completing the SQ-FFQ, to aid the estimation of portion size intake [22]. All information provided by the participants was double-checked for accuracy. The recorded data was analyzed with the Indonesian Food Database and Nutrisurvey (EBISpro, Germany) to estimate energy as well as macro- and micronutrient consumption. Wherever appropriate, nutrient intake values were adjusted to energy by the nutrient (energy-adjusted) residual method [23].

“The Global Physical Activity Questionnaire” (GPAQ), developed by the World Health Organization (WHO) was used to measure physical activity [24]. Total time in moderate-to-vigorous physical activity was calculated according to the WHO STEPwise method and was expressed as metabolic equivalent minutes per day (METmins/day). Furthermore, participants were classified as “active” if they accumulated ≥600 METmins/week or “inactive” if they did <600 METmins/week. Sedentary behaviour (SB, mins/day) was determined from the last question of the GPAQ, based on how long the participants spent sitting while working, in a vehicle, watching television, or lying down, except sleeping [24].

### SNP selection and genotyping

We selected nine vitamin B12-related SNPs (Methylenetetrahydrofolate reductase [*MTHFR*]- rs1801133, Carbamoyl-phosphate synthase 1 [*CPS1*]- rs1047891, Cubulin [*CUBN*]- rs1801222, CD320 molecule [*CD320*]- rs2336573, Transcobalamin 2 [*TCN2*]- rs1131603, Fucosyltransferase 2 [*FUT2*]- rs602662, Transcobalamin 1 [*TCN1*]- rs34324219, Fucosyltransferase 6 [*FUT6*]- rs778805 and Methylmalonyl-CoA mutase [*MUT*]- rs1141321) based on the recent review article by Surendran et al. [18].

The nine metabolic disease-related SNPs were selected for our analysis based on previously published candidate gene association and genome-wide association (GWA) studies for metabolic disease-related traits [25–33]: Fat mass and obesity-associated [*FTO*]- rs9939609 and rs8050136, Melanocortin 4 Receptor [*MC4R*]- rs17782313 and rs2229616, Transcription factor 7-like 2 [*TCF7L2*]- rs12255372 and rs7903146, Potassium voltage-gated channel subfamily J member 11 [*KCNJ11*]- rs5219, Calpain 10 [*CAPN10*]- rs3792267 and rs5030952).

Genomic DNA was isolated from peripheral blood leukocytes using the PureLink Genomic DNA Mini Kit (Invitrogen, Carlsbad, USA) with spin column methods. The DNA concentration was determined using a NanoDrop spectrophotometer. Genotyping was performed at LGC Genomics (http://www.lgcgroup.com/services/genotyping), which employs the competitive allele-specific PCR-KASP® assay.

### Statistical analysis

The SPSS statistical package (version 22; SPSS Inc., Chicago, IL, USA) was used for the statistical analysis. Results from the descriptive analyses are presented as means and standard deviations (SD) for continuous variables and as percentages for categorical variables. Generalized obesity was defined according to the Asia-Pacific classification of BMI for Asians as non-obese (BMI < 25 kg/m^2^) and obese (BMI ≥ 25 kg/m^2^) [21]. We performed an independent t-test to compare the means of the quantitative variables between non-obese individuals vs obese individuals. Comparison of the proportion of individuals engaging in different types of physical activity levels (vigorously active, moderately active and sedentary) between non-obese individuals vs obese individuals was analyzed by the Chi Square test. The normality of variable distribution was verified by the Shapiro-Wilk test; WC, body fat percentage, glucose, insulin, HbAC1 and vitamin B12 levels were not normally distributed in our study population; therefore, the data were natural log-transformed prior to analysis.

Allele frequencies were estimated by gene counting. The Chi-square test was used to compare the proportions of genotypes or alleles. Fifteen of the SNPs were in Hardy Weinberg Equilibrium (HWE) (*P* > 0.05) (Table 1). HWE was not calculated for the SNPs *TCN2* rs1131603 and *TCN1* rs34324219 as no minor alleles were present. The SNP *FUT2* rs602662 deviated from HWE; however, this SNP was not excluded from analysis. The KASP™ genotyping technology used in this study, has been independently assessed to be over 99.8% accurate [34]. Validation of the KASP™ genotyping was conducted at LGC genomics and the quality of the genotyping results were independently assessed and confirmed by the project manager. This ruled out genotyping artefacts as possible reasons for deviation from HWE. Hence, it is possible that the SNP *FUT2* rs602662 could have deviated from HWE due to population or racial grouping substructure (Sub-grouping), non-random mating, linkage disequilibrium (incomplete mixing of different ancestral population) or chance findings [35].

**Table 1:** Genotype distribution of vitamin B12 related SNPs and metabolic disease-related SNPs

Gene	rs number	Major allele	Minor allele	Common Homozygotes (%)	Heterozygotes (%)	Rare Homozygotes (%)	Minor allele frequency	HWE P value
*MTHFR*	*rs1801133*	C	T	92 (79.30)	24 (20.70)	0 (0.00)	0.10	0.214
*CPS1*	*rs1047891*	C	A	48 (41.00)	56 (47.90)	13 (11.10)	0.35	0.579
*CUBN*	*rs1801222*	C	T	84 (74.30)	27 (23.90)	2 (1.80)	0.14	0.920
*CD320*	*rs2336573*	C	T	86 (74.10)	29 (25.00)	1 (0.90)	0.13	0.390
*TCN2*	*rs1131603*	T	C	117 (100)	0 (0.00)	0 (0.00)	0	N/A
*FUT2*	*rs602662*	G	A	111 (94.90)	4 (3.40)	2 (1.70)	0.03	0.000
*TCN1*	*rs34324219*	C	A	117 (100)	0 (0.00)	0 (0.00)	0	N/A
*FUT6*	*rs778805*	T	C	33 (28.20)	61 (52.10)	23 (19.70)	0.46	0.586
*MUT*	*rs1141321*	G	A	67 (59.30)	40 (35.40)	6 (5.30)	0.23	0.993
*CAP10*	*rs3792267*	G	A	108 (91.50)	9 (7.60)	1 (0.80)	0.05	0.123
*CAP10*	*rs5030952*	C	T	77 (66.40)	31 (26.70)	8 (6.90)	0.20	0.063
*KCNJ11*	*rs5219*	C	T	55 (47.00)	47 (40.20)	15 (12.80)	0.33	0.329
*TCF7L2*	*rs12255372*	G	T	97 (82.90)	20 (17.10)	0 (0.00)	0.09	0.312
*TCF7L2*	*rs7903146*	C	T	95 (81.90)	21 (18.10)	0 (0.00)	0.09	0.284
*FTO*	*rs9939609*	T	A	70 (60.30)	39 (33.60)	7 (6.00)	0.23	0.618
*MC4R*	*rs17782313*	T	C	89 (76.10)	26 (22.20)	2 (1.70)	0.13	0.929
*FTO*	*rs8050136*	C	A	69 (60.00)	39 (33.90)	7 (6.10)	0.23	0.638
*MC4R*	*rs2229616*	G	A	116 (99.10)	1 (0.90)	0 (0.00)	0.00	0.963

*MAF* minor allele frequency, *HWE* Hardy Weinberg Equilibrium, *X*^*2*^ Chi-Squared value

**Fig. 2:**
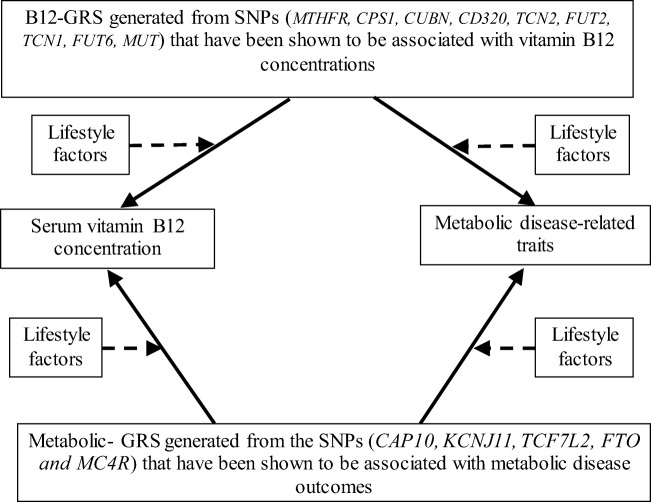
Diagram representing the study design. Four possible associations, and four possible interactions were examined. One-sided arrows with unbroken lines represent genetic associations and one-sided arrows with broken lines represent interactions between a GRS and a lifestyle factor on serum vitamin B12/ metabolic traits (such as glucose, insulin, HbA1c, BMI, WC and body fat percentage). The association of the metabolic-GRS with vitamin B12 concentrations and metabolic disease-related traits and the association of B12 –GRS with vitamin B12 concentrations and metabolic disease related traits were tested. Lastly, the impact of lifestyle factors (macronutrient intakes and physical activity levels) on these genetic associations was investigated

A schematic representation of the study design is presented in Fig. 2. The unweighted, risk-allele GRS method was calculated for each participant as the sum of risk allele counts across each SNP which predicted vitamin B12 status. The B12-GRS was generated from the vitamin B12-related SNPs in the *MTHFR, CPS1, CUBN, CD320, TCN2, FUT2, TCN1, FUT6, MUT* genes. Furthermore, another unweighted GRS was created using allele markers previously reported to be associated with metabolic disease traits. The Metabolic-GRS was generated from the SNPs in the *CAP10*, *KCNJ11*, *TCF7L2*, *FTO* and *MC4R* genes. A value of 0, 1 or 2 was assigned to each SNP, which denotes the number of risk alleles on that SNP. These values were then calculated by adding the number of risk alleles across each SNP. The average number of risk alleles per person for the B12-GRS was 8.18 (SD = 1.36), which ranged from 5 to 12. The sample was stratified, by the median, into a “low genetic risk group,” for those with a GRS ≤ 8 risk alleles (*n* = 73), and into a “high genetic risk group,” for those with a GRS ≥ 9 risk alleles (*n* = 44). For the metabolic-GRS, the average number of risk alleles per person was 4.66 (SD = 1.76), which ranged from 2 to 9. The sample was stratified, by the median, into a “low genetic risk group,” for those with a GRS ≤ 4 risk alleles (*n* = 61), and into a “high genetic risk group,” for those with a GRS ≥ 5 risk alleles (*n* = 56). Linear regression was used to examine the association of the two GRS scores with the biochemical and anthropometric outcomes (vitamin B_12_, glucose, insulin, HbA1c, BMI, WC and body fat percentage). The interaction between the two GRS scores and dietary factors on biochemical and anthropometric outcomes was determined by including interaction term (GRS*lifestyle factor) in the regression model. Models were adjusted for age, BMI, and total energy intake, wherever appropriate. Correction for multiple testing was applied using Bonferroni correction [2 GRS * 7 biochemical and anthropometric measurements (vitamin B12, glucose, insulin, HbA1c, BMI, WC, body fat percentage) * 5 lifestyle factors (dietary carbohydrate (energy %), dietary protein (energy %), dietary fat (energy %), dietary fibre (g) and physical activity levels) = 70 tests; 0.05/70 = 0.000714; *P* < 0.000714]. All data are expressed as mean ± SD. Given that there are no studies on GRS in relation to B12 status and metabolic outcomes and no previously reported effect sizes for the South-East Asians, we were unable to perform a power calculation.

## Results

### Characteristics of the participants

In this study, 117 women (mean age, 40.30 ± 10.10 years; BMI, 25.10 ± 4.20 kg/m^2^) were included. Table 2 illustrates the main characteristics of the study participants.

**Table 2:** Anthropometric and biochemical characteristics of women participants

	All women	Non-obese*	Obese**	P value***
	(N = 117)	(*N* = 32)	(*N* = 85)	
Age (yrs)	40.40 ± 10.20	35.70 ± 11.30	42.10 ± 9.20	0.006
Height (cm)	152.90 ± 5.20	154.90 ± 4.70	152.20 ± 5.20	0.012
BMI (kg/m^2^)	25.10 ± 4.20	20.10 ± 2.10	27.00 ± 3.10	<0.001
WC (cm)	83.10 ± 12.50	72.80 ± 13.30	87.00 ± 9.70	<0.001
Body fat (%)	35.70 ± 7.00	27.00 ± 5.20	39.00 ± 4.30	<0.001
Fasting serum Glucose (mg/dl)	92.20 ± 20.20	85.70 ± 9.00	94.70 ± 22.70	0.033
Fasting serum Insulin (mIU/L)	32959 ± 26327	30372 ± 26179	33933 ± 26470	0.517
HbA1C (ng/ml)	662 ± 624	638 ± 606	672 ± 633	0.794
Fasting vitamin B12 (pg/mL)	591 ± 579	426 ± 137	433 ± 193	0.795
Physical Activity Levels	Sedentary (39.30%)	Sedentary (46.90%)	Sedentary (36.50%)	0.490^a^
	Moderate (49.60%)	Moderate (40.60%)	Moderate (52.90%)	
	Vigorous (11.10%)	Vigorous (12.50%)	Vigorous (10.60%)	
Total energy (kcal/d)	1774 ± 609	1849 ± 585	1746 ± 619	0.416
Protein (g)	76.90 ± 36.50	80.50 ± 29.00	75.50 ± 39.00	0.514
Fat (g)	59.00 ± 33.10	67.30 ± 27.70	55.80 ± 34.60	0.096
Carbohydrate (g)	233 ± 71	230 ± 70	235 ± 72	0.714
Dietary fibre (g)	8.80 ± 4.50	9.70 ± 4.80	8.50 ± 4.40	0.222
Saturated Fat (g)	20.90 ± 11.10	23.70 ± 11.10	19.80 ± 10.90	0.085
MUFA (g)	8.20 ± 4.50	9.80 ± 5.20	7.50 ± 4.20	0.015
PUFA (g)	6.30 ± 3.50	6.80 ± 3.20	6.10 ± 3.60	0.332

Data shown are represented as means ± SD

P values were calculated by using the Independent t test

Abbreviations: BMI Body mass index; WC Waist circumference; MUFA Monounsaturated fatty acids; PUFA Polyunsaturated fatty acids

*Non-Obese individuals refers to the percentage of individuals with a BMI of under 23 according to the Asia-Pacific classification of BMI

**Obesity cases refers to the percentage of individuals with a BMI of equal to or over 23 according to the Asia-Pacific classification of BMI

***P values for the differences in the means/ proportions between non-obese and obese individuals

^a^P values were calculated by using the Chi Squared test

### Association between B12-GRS and metabolic-GRS with biochemical and anthropometric measurements

After correction for multiple testing, none of the associations of the B12-GRS with vitamin B12 and metabolic traits (*P* > 0.160) were statistically significant (Supplementary Table 1). Furthermore, no associations between the metabolic-GRS and vitamin B12 or metabolic traits (*P* > 0.085) were observed (Supplementary Table 2).

### Interaction between the B12-GRS and dietary factors on biochemical and anthropometric measurements

**Fig. 3:**
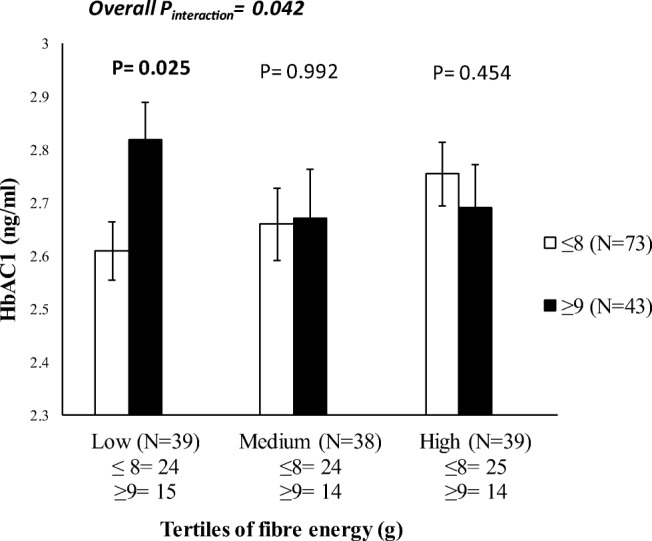
Interaction between the B12-GRS and dietary fibre intake (g) on log HbAC1 (ng/ml) (P_interaction_ = 0.042). Among those who consumed a low fibre diet, individuals who carried 9 or more risk alleles had significantly higher levels of log HbAC1 compared to individuals carrying 8 or less risk alleles (*P* = 0.025)

We observed an interaction between the B12-GRS and dietary fibre intake (g) on log transformed HbA1C (P_interaction_ = 0.042) (Fig. 3 and Supplementary Table 3). Individuals who carried 9 or more risk alleles for vitamin B12 deficiency had 8.10% higher HbAC1 concentrations (ng/ml) in the lowest tertile of fibre intake (g) (Mean ± S.D.: 4.90 ± 1.00 g) compared to those with 8 or less risk alleles for vitamin B12 deficiency.

Interactions were also seen between the B12-GRS and protein (energy %) on log transformed body fat percentage (*P* = 0.034). However, further stratification of participants based on their consumption of low-, medium- and high-dietary protein (energy %) did not show a statistically significant association between the GRS and the outcomes in any of the tertiles.

### Interaction between the metabolic-GRS and dietary factors on biochemical and anthropometric measurements

**Fig. 4:**
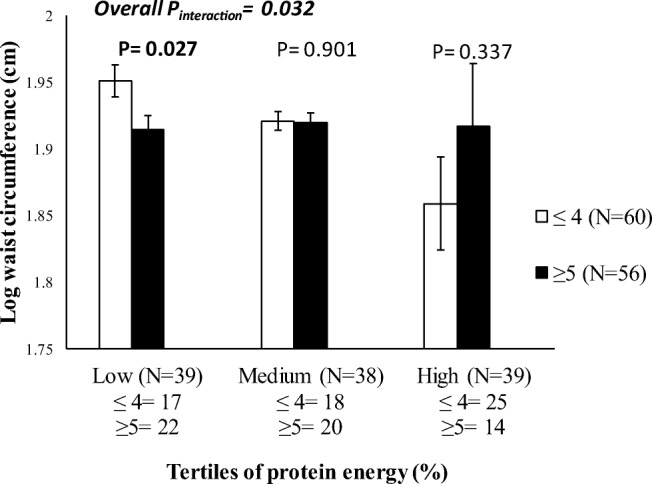
Interaction between the metabolic-GRS and protein energy (%) on log waist circumference (P_interaction_ = 0.032). Among those who consumed a low protein diet, individuals who carried 5 or more risk alleles had significantly lower waist circumference measurements compared to individuals carrying 4 or less risk alleles (*P* = 0.027)

An interaction was found between the metabolic-GRS and protein (energy %) on log transformed WC (*P* = 0.032) (Supplementary Table 3 and Fig. 4). Individuals who carried 5 or more risk alleles for metabolic disease had 2.15% lower WC measurements (cm) in the lowest tertile of protein energy intake (%) (Mean ± S.D.: 1.91 ± 0.06%) compared to those with 4 or less risk alleles (*P* = 0.027) (Fig. 4).

### Interaction between the B12-GRS and physical activity on biochemical and anthropometric measurements

No statistically significant interactions were observed between the two GRSs (vitamin B12 and metabolic traits) and physical activity on biochemical and anthropometric measurements (*P* > 0.056) (Supplementary Table 3)**.**

After correction for multiple testing (Bonferroni corrected <0.000714), none of these GRS-lifestyle interactions were considered statistically significant.

## Discussion

To our knowledge, this is the first study to use a nutrigenetic approach to explore the relationship between vitamin B2 status and metabolic traits in Indonesian women. Our study demonstrated the impact of genetically instrumented B12 concentrations on HbA1C levels, a marker of glycaemic control [36], through the influence of dietary fibre intake. Given that previous studies have shown that the consumption of dietary fibre is inadequate in Indonesian adults [37–39], our findings, if replicated in future studies, may have significant public health implications in terms of encouraging a consumer education campaign targeted around increasing fibre intake, in order to reduce HbA1C levels, which may be associated with improved glycaemic control.

In the present study, we constructed a GRS consisting of nine vitamin B12 decreasing SNPs in genes involved in vitamin B12 metabolism [18]. Our study showed that individuals carrying less than 8 risk alleles for vitamin B12 deficiency had higher vitamin B12 concentrations, compared to those carrying more than 9 risk alleles. However, there was no statistically significant difference between individuals carrying 8 or less risk alleles vs 9 or more risk alleles for the B12-GRS, which could be attributed to the small sample size. Furthermore, we were unable to identify any association between the B12-GRS and metabolic disease traits in our study, implying that linear decreases in vitamin B12 may not have a role in the development of metabolic disease traits. Our finding goes in line with a Mendelian Randomization study investigating the effect of genetically instrumented vitamin B12 concentrations on BMI, where there was no evidence to suggest the causal role of decreased serum vitamin B12 levels in obesity [40].

Interestingly, in our study, we found a significant interaction between the B12-GRS and dietary fibre intake (g) on log HbA1C levels, where, among those who consumed a low fibre diet (Mean ± S.D.: 13.60 ± 4.30), individuals carrying more than 9 risk alleles had significantly higher HbAC1 levels compared to those carrying 8 or less risk alleles. The average fibre intake in Indonesia is 10.5 g/day [41], which is lower compared to the mean fibre intake in the UK (~18 g/day) and USA (~16 g/day) [28]. In comparison to the mean fibre intake in Indonesia, the results in our study reported a lower mean fibre intake (8.80 ± 4.50 g). It is important that dietary intakes of fibre are increased in this population, as it may help maintain lower levels of HbAC1 levels in individuals carrying more than 9 risk alleles of the B12-GRS. Even though our study is the first to report this gene-diet interaction, a meta-analysis conducted from 15 randomized studies have shown that high fibre intake can reduce HbAC1 levels in type 2 diabetic subjects [42]. High fibre intake is generally recommended to reduce the risk of gestational diabetes (GDM) in pregnant women [43]. It has been shown that each 10 g/day increment in total fibre intake, corresponds to a 26% reduced risk of GDM [43]. It is possible that high dietary fibre may increase satiety and consequently reduce total energy intake [44, 45]. Increased dietary fibre intake may also affect glucose homeostasis, by delaying gastric emptying, resulting in a slower absorption of glucose into the blood stream [43]. Additionally, low vitamin B12 status prevents erythropoiesis and prolongs the lifespan of erythrocytes, resulting in increased HbA1c levels [46]. This is the first study to provide evidence for an interaction between B12-GRS and HbA1c, hence, we do not have any previous studies to compare our findings with.

Accurately determining obesity has become an exceedingly important step in preventing the onset of metabolic syndrome or cardiometabolic diseases, which are brought about through excess adiposity. The underestimation of obesity, particularly in young women who appear to have a healthy BMI measure, could falsely lead to incorrect conclusions about body composition and future risk of diseases associated with increased adiposity, such as breast cancer [47]. The ability to measure body fat percentage is currently the preferred method of determining body composition over BMI, as it distinguishes between fat and lean body mass [48]. To date, little is known about the average body fat percentage in healthy Indonesian women. Although, a recent study conducted in 308 Indonesian women of Javanese ethnicity living in Yogyakarta Special Region Province (aged between 18 and 65), reported lower body fat percentage values (33.30 ± 7.70%) compared to our present study (35.70 ± 7.00%) [49]. Within our study, an interaction between the B12-GRS and protein intake (energy %) on log transformed body fat percentage was observed. The exact mechanism of how dietary protein results in a more favourable body composition profile in individuals genetically predisposed to vitamin B12 deficiency is not known. A previous study conducted in 1,834 participants in Canada reported that high protein diets could reduce overall body fat percentage, even in the absence of energy restriction [50]. Further to this, it has been hypothesised that high protein diets increase the release of the anorectic gut hormone peptide YY (PYY), thus enhancing greater inter-meal satiety and reducing weight gain [51]. This suggestion for a novel interaction of protein (energy intake) in relation to body fat distribution in individuals genetically predisposed to vitamin B12 deficiency warrants further replication.

In a review analysing the nutrient intakes of pregnant women in Indonesia, it was reported that pregnant Indonesian women generally have protein intakes below the estimated average requirements [52]. The association between low protein intake and obesity outcomes has attracted interest amongst health care professionals. Observational studies in the USA have reported that body weight and WC were reduced when protein was consumed above the recommended daily allowance [0.8 g/kg body weight (BW)] [53]. It has been noted in animal models, that pregnant rats consuming a low protein diet were more prone to GDM and to having offspring with a low birthweight [54, 55]. In our study, we found a significant interaction between the metabolic-GRS and protein energy (%) on log WC, where individuals consuming a low protein diet, despite carrying 5 or more risk alleles, had a lower waist to hip ratio compared to individuals carrying 4 or less risk alleles. There are no previous reports of the risk variants used in our GRS, but Goni et al. [36] found that total protein intake interacted with a GRS of 16 obesity/lipid metabolism polymorphisms to modify the effect on body fat mass in 711 individuals of Caucasian ancestry. In our study, we only observed interaction of the metabolic-GRS with WC but not BMI, which suggests that effects of the GRS are likely to be on central obesity as opposed to common obesity in Indonesian women.

Significant interactions between genetic variants and physical activity on obesity traits have been reported in several studies from Europe and Asia [56, 57]. However, this is the first study to investigate interactions between the two GRSs and physical activity on metabolic traits and B12 concentrations in Indonesian women. In our study, as much as 39% of the women had low physical activity levels. These findings were much higher than the findings reported from another cross sectional study conducted across five major cities in Indonesia, who reported that 20% of women had a low physical activity status [58]. Although the majority of women in our study were physically inactive, no significant interactions were found between the GRSs and physical activity on metabolic traits/B12 status, which could be due to the small sample size of our study.

Major strengths of our study are that this is the first study of its kind to evaluate vitamin B12 status among Indonesian women. Furthermore, this study used a comprehensive, validated, interviewer administered food frequency questionnaire [20] to measure the long-term macronutrient intake of the population. Nevertheless, several limitations of this study need to be considered. One of the main limitations of the study is the small sample size (*N* = 117); however, we were still able to identify significant associations and gene-lifestyle interactions. Given that the study used a SQ-FFQ, self-reported data might have biased the dietary intake information. The study had included data on total energy and macronutrient intake but no data on specific types of foods or micronutrients were included. This limited the analysis of testing for interaction of specific food components with GRS on various outcome measures. Circulating concentrations of other vitamin B12 biomarkers, such as Holo-transcobalamin (holoTC) or Methylmalonic Acid (MMA) were not measured. Furthermore, all the women included in our analysis were of Minangkabau descent, and thus it is unknown whether our results can be generalized to other communities in Indonesia.

In conclusion, our study showed a significant effect of the B12-GRS on HbA1C concentrations, through the influence of a low dietary fibre intake. Additionally, our study failed to provide evidence for an impact of metabolic-GRS on lowering B12 concentrations. After correction for multiple testing, none of the interactions were statistically significant; hence, further replication studies utilizing larger sample sizes are needed to confirm our findings, before public health recommendations and personalized nutrition advice can be developed for Minangkabau Indonesian women.

## Electronic supplementary material

ESM 1 (DOCX 22 kb)

## Data Availability

Data from this project will not be shared because additional results from the study are yet to be published.

## Abbreviations

SNPs: single nucleotide polymorphisms
*MTHFR*: Methylenetetrahydrofolate reductase
*CPS1*: Carbamoyl-phosphate synthase 1
*CUBN*: Cubulin
*CD320*: CD320 molecule
*TCN2*: Transcobalamin 2
*FUT2*: Fucosyltransferase 2
*TCN1*: Transcobalamin 1
*FUT6*: Fucosyltransferase 6
*MUT*: Methylmalonyl-CoA mutase
*CAP10*: Calpain 10
*KCNJ11*: Potassium voltage-gated channel subfamily J member 11
*TCF7L2*: Transcription factor 7-like 2
*FTO*: Fat mass and obesity-associated
*MC4R*: Melanocortin 4 Receptor
BMI: body mass index
SD: indicates standard deviations
WC: waist circumference
