# Supplementary material for: A nutrigenetic approach for investigating the relationship between vitamin B12 status and metabolic traits in Indonesian women
Source: J Diabetes Metab Disord. 2019 Jul 25;18(2):389–99. doi: 10.1007/s40200-019-00424-z (PMC6914754; doi:10.1007/s40200-019-00424-z)
Supplement: Supplementary file 1 — (DOCX 22 kb) [file 40200_2019_424_MOESM1_ESM.docx]

**Supplementary Table 1:** **Association of the B12-GRS with obesity traits, biochemical traits and anthropometric measurements**

| **GRS** | **BMI (kg/m^2^)** | **WC (cm)** | **Body Fat (%)** | **Fasting serum glucose (mg/dl)** | **Fasting serum insulin (mIU/L)** | **HbAC1 (ng/ml)** | **Vitamin B12 (pg/ml)** |
| --- | --- | --- | --- | --- | --- | --- | --- |
| ≤ 8 risk alleles | 24.90 ± 4.10 | 82.10 ± 13.20 | 35.40 ± 7.10 | 89.90 ± 9.30 | 31331 ± 24636 | 625 ± 579 | 452 ± 187 |
| ≥ 9 risk alleles | 25.50 ± 4.30 | 84.60 ± 11.00 | 36.30 ± 6.90 | 96.10 ± 30.60 | 35659 ± 29008 | 726 ± 693 | 392 ± 156 |
| P value | 0.468† | 0.456* | 0.898* | 0.193* | 0.328* | 0.444* | 0.160* |

Values are given as mean ± standard deviation.

P values for differences between ≤8 and ≥9 risk alleles were obtained using linear regression model adjusted for age, sex and BMI.

^†^ P values were obtained by using a general linear model adjusted for age and sex

*P values were based on the log transformed values

Abbreviations: GRS, Genetic risk score; BMI, body mass index; WC, waist circumference; HbA1C, glycated haemoglobin

**Supplementary Table 2:** **Association of the metabolic-GRS with obesity traits and biochemical and anthropometric measurements**

| GRS | BMI (kg/m^2^) | WC (cm) | Body Fat (%) | Fasting serum glucose (mg/dl) | Fasting serum insulin (mIU/L) | Glycated Haemoglobin (ng/ml) | Vitamin B12 (pg/ml) |
| --- | --- | --- | --- | --- | --- | --- | --- |
| ≤ 4 risk alleles | 24.50 ±4.10 | 82.30 ± 14.40 | 35.00 ± 6.80 | 95.00 ± 26.00 | 33764 ± 27805 | 670 ± 651 | 436 ± 174 |
| ≥ 5 risk alleles | 25.80 ± 4.20 | 83.90 ± 10.20 | 36.50 ± 7.20 | 89.20 ± 10.30 | 32082 ± 24837 | 654 ± 598 | 426 ± 184 |
| P value | 0.085^†^ | 0.570* | 0.383* | 0.361* | 0.785* | 0.653* | 0.778* |

Values are given as mean ± standard deviation

P values for differences between ≤4 and ≥5 risk alleles were obtained using linear regression model adjusted for age, sex and BMI.

^†^ P values were obtained by using a general linear model adjusted for age and sex

*P values were based on the log transformed values

Abbreviations: BMI body mass index; WC waist circumference; HbA1C, glycated haemoglobin

**Supplementary Table 3:** **Interaction between the B12-GRS and metabolic-GRS and lifestyle factors on biochemical outcomes and anthropometric measurements**

| ***Interaction between the GRS * lifestyle factors on BMI*** | | | | |
| --- | --- | --- | --- | --- |
| B12-GRS * fat energy % | B12-GRS * carbohydrate energy % | B12-GRS * protein energy % | B12-GRS * fibre (g) | B12-GRS * Physical activity levels |
| 0.933† | 0.685† | 0.993† | 0.155† | (0.682†) |
| metabolic-GRS * fat energy % | metabolic-GRS * carbohydrate energy % | metabolic-GRS * protein energy % | metabolic-GRS * fibre (g) | metabolic-GRS * Physical activity levels |
| 0.422† | 0.230† | 0.110† | 0.273† | 0.757† |
| ***Interaction between the GRS **** ***lifestyle factors on Log waist circumference (cm)*** | | | | |
| B12-GRS * fat energy % | B12-GRS * carbohydrate energy % | B12-GRS * protein energy % | B12-GRS * fibre (g) | B12-GRS * Physical activity levels |
| 0.444 | 0.875 | 0.395 | 0.547 | 0.706 |
| metabolic-GRS * fat energy % | metabolic-GRS * carbohydrate energy % | metabolic-GRS * protein energy % | metabolic-GRS * fibre (g) | metabolic-GRS * Physical activity levels |
| 0.812 | 0.072 | **0.032** | 0.648 | 0.796 |
| ***Interaction between the GRS **** ***lifestyle factors on Log Body fat (%)*** | | | | |
| B12-GRS * fat energy % | B12-GRS * carbohydrate energy % | B12-GRS * protein energy % | B12-GRS * fibre (g) | B12-GRS * Physical activity levels |
| 0.275 | 0.064 | **0.034** | 0.697 | 0.419 |
| metabolic-GRS * fat energy % | metabolic-GRS * carbohydrate energy % | metabolic-GRS * protein energy % | metabolic-GRS * fibre (g) | metabolic-GRS * Physical activity levels |
| 0.775 | 0.844 | 0.568 | 0.423 | 0.253 |
| ***Interaction between the GRS * lifestyle factors on Log fasting serum glucose (mg/dl)*** | | | | |
| B12-GRS * fat energy % | B12-GRS * carbohydrate energy % | B12-GRS * protein energy % | B12-GRS * fibre (g) | B12-GRS * Physical activity levels |
| 0.347 | 0.260 | 0.368 | 0.380 | 0.315 |
| metabolic-GRS * fat energy % | metabolic-GRS * carbohydrate energy % | metabolic-GRS * protein energy % | metabolic-GRS * fibre (g) | metabolic-GRS * Physical activity levels |
| 0.634 | 0.771 | 0.929 | 0.537 | 0.056 |
| ***Interaction between the GRS * lifestyle factors on Log fasting serum insulin (mIU/L)*** | | | | |
| B12-GRS * fat energy % | B12-GRS * carbohydrate energy % | B12-GRS * protein energy % | B12-GRS * fibre (g) | B12-GRS * Physical activity levels |
| 0.757 | 0.341 | 0.073 | 0.215 | 0.629 |
| metabolic-GRS * fat energy % | metabolic-GRS * carbohydrate energy % | metabolic-GRS * protein energy % | metabolic-GRS * fibre (g) | metabolic-GRS * Physical activity levels |
| 0.108 | 0.104 | 0.890 | 0.947 | 0.723 |
| ***Interaction between the GRS * lifestyle factors on Log HbAC1 (ng/ml)*** | | | | |
| B12-GRS * fat energy % | B12-GRS * carbohydrate energy % | B12-GRS * protein energy % | B12-GRS * fibre (g) | B12-GRS * Physical activity levels |
| 0.175 | 0.091 | 0.150 | **0.042** | 0.475 |
| metabolic-GRS * fat energy % | metabolic-GRS * carbohydrate energy % | metabolic-GRS * protein energy % | metabolic-GRS * fibre (g) | metabolic-GRS * Physical activity levels |
| 0.298 | 0.166 | 0.155 | 0.851 | 0.969 |
| ***Interaction between the GRS * lifestyle factors on Log Vitamin B12 (pg/ml)*** | | | | |
| B12-GRS * fat energy % | B12-GRS * carbohydrate energy % | B12-GRS * protein energy % | B12-GRS * fibre (g) | B12-GRS * Physical activity levels |
| 0.772 | 0.936 | 0.270 | 0.157 | 0.078 |
| metabolic-GRS * fat energy % | metabolic-GRS * carbohydrate energy % | metabolic-GRS * protein energy % | metabolic-GRS * fibre (g) | metabolic-GRS * Physical activity levels |
| 0.983 | 0.682 | 0.298 | 0.171 | 0.242 |

P values were obtained by using a general linear model adjusted for age, sex, and BMI

† P values were obtained by using a general linear model adjusted for age and sex

Abbreviations: GRS, Genetic risk score; BMI, body mass index
